# Patient Characteristics and Outcomes of Acute Pancreatitis Managed in a Hospital-at-Home Program: A Multicenter Retrospective Descriptive Study

**DOI:** 10.3390/healthcare14182919

**Published:** 2026-09-09

**Authors:** Tatjana Gavrancic, Igor Dumic, Jessica Laenger, Brittany S. Jackson, Khanyisile N. Tshabalala, Michele D. Lewis, Margaret R. Paulson, Michael J. Maniaci, Wendelyn Bosch

**Affiliations:** 1 Division of Hospital Internal Medicine, Mayo Clinic, Jacksonville, FL 32224, USA; michael.maniaci@advocatehealth.org; 2 Division of Hospital Internal Medicine, Mayo Clinic Health System, Eau Claire, WI 54703, USA; dumic.igor@mayo.edu (I.D.); paulson.margaret@mayo.edu (M.R.P.); 3 Division of Hospital Medicine, Department of Medicine, Lahey Clinic, Burlington, MA 01805, USA; 4 Division of Community Internal Medicine, Mayo Clinic College of Medicine and Science, Jacksonville, FL 32224, USA; laenger.jessica@mayo.edu (J.L.); britt1jack@hotmail.com (B.S.J.); 5 Department of Research, Mayo Clinic, Jacksonville, FL 32224, USA; yrh8hc@virginia.edu; 6 Division of Gastroenterology and Hepatology, Mayo Clinic, Jacksonville, FL 32224, USA; lewis.michele@mayo.edu; 7 Division of Infectious Diseases, Mayo Clinic, Jacksonville, FL 32224, USA; bosch.wendelyn@mayo.edu

**Keywords:** hospital-at-home, acute pancreatitis, advanced care at home, virtual hybrid care, clinical outcomes

## Abstract

**Background/Objectives:** Hospital-at-home (HaH) programs have emerged as an alternative model to brick-and-mortar (BaM) hospital management of acute conditions such as heart failure, pneumonia, pyelonephritis, and chronic obstructive pulmonary disease exacerbation among the others. However, the clinical management of acute pancreatitis in the HaH setting has never been described in the literature. The primary objective of this study was to describe the demographic and clinical characteristics of patients with acute pancreatitis managed in a Mayo Clinic Advanced Care at home (ACH) program. The secondary objective was to describe feasibility and short-term clinical outcomes. **Methods:** This multistate, multicenter, retrospective descriptive study included 40 unique patients with acute pancreatitis managed at ACH between 13 October 2020 and 1 June 2024. Repeat admissions were excluded from the primary analysis. **Results:** The most common etiologies were idiopathic (42.5%), gallstone-related (22.5%), and alcohol-related (12.5%). Peripancreatic fluid collections occurred in 17.5%, necrotizing pancreatitis in 10.0%, pleural effusion in 12.5%, and infected pancreatitis in 7.5%. Intravenous antiemetics were administered to 22.5%, intravenous fluids to 62.5%, and intravenous opioids to 7.5%. Median total hospital length of stay was 5.0 days (IQR, 3.0–8.25), including a median of 3.0 days (IQR, 2.0–4.0) in ACH. Four patients (10.0%; exact 95% CI, 2.8–23.7%) were transferred from ACH to BaM care. Seven-day and 30-day readmission rates were 10% (95% CI: 2.8%, 23.7%) and 5% (95%CI: 0.6%, 16.9%), respectively. The 30-day emergency department visit was 0%. Although 30-day mortality was 2.5% (95% CI: 0.0%, 13.2%), in-program mortality was 0%. **Conclusions:** Among a highly selected group of clinically stable patients meeting the program’s eligibility criteria, management of acute pancreatitis in ACH was feasible and was associated with low observed rates of unplanned escalations and short-term adverse outcomes.

## 1. Introduction

Acute pancreatitis presents a substantial healthcare burden and is commonly managed with supportive care including fluid resuscitation, analgesia, antiemetics, and nutritional support [1,2]. Traditional inpatient management is resource intensive [3]. With the introduction of the Centers for Medicare & Medicaid Services (CMS) reimbursement pathway for hospital-at-home (HaH) in 2020, some healthcare systems in the U.S. started offering inpatient care in the comfort of patient’s home as an alternative to institutional hospital settings [4]. HaH allows health systems to address the growing healthcare needs of communities by accommodating increasing demand for acute care without the need to increase the physical space of a hospital. Randomized studies in selected acute medical populations have reported comparable short-term outcomes between HaH and conventional hospitalization [5,6]. HaH has been primarily utilized to treat a narrow range of common conditions such as pneumonia, chronic obstructive pulmonary disease and asthma exacerbation, heart failure, diverticulitis and pyelonephritis [4,7,8].

Acute pancreatitis treatment and outcomes in traditional brick and mortar (BaM) hospitalization have been described extensively, but there is a lack of literature on feasibility and clinical outcomes of acute pancreatitis treated at HaH [1,9,10]. The primary aim of this study was to describe the demographic and clinical characteristics of an acute pancreatitis cohort managed in HaH. The secondary aim was to describe feasibility and safety outcomes.

## 2. Materials and Methods

### 2.1. Study Setting

The Mayo Clinic’s HaH program, Advanced Care at Home (ACH), is a virtual hybrid model of inpatient care delivery managed remotely by hospitalist physicians, advanced practice providers (APPs: licensed clinicians such as nurse practitioners and physician assistants), and registered nurses (RNs) in a centralized command center. This team partners with in-home personnel that includes community paramedics, home health RNs, and other external home care and delivery vendors through an integrated healthcare network, ensuring the needed services are delivered to the patient in the home [11]. The ACH program utilized DispatchHealth (Boston, MA, USA) for care orchestration, in-home equipment, and video conferencing infrastructure; Epic Systems Corporation (Verona, WI, USA) for the electronic health record platform; and Acadian Ambulance (Lafayette, LA, USA) as the in-home care vendor.

**Figure 1:**
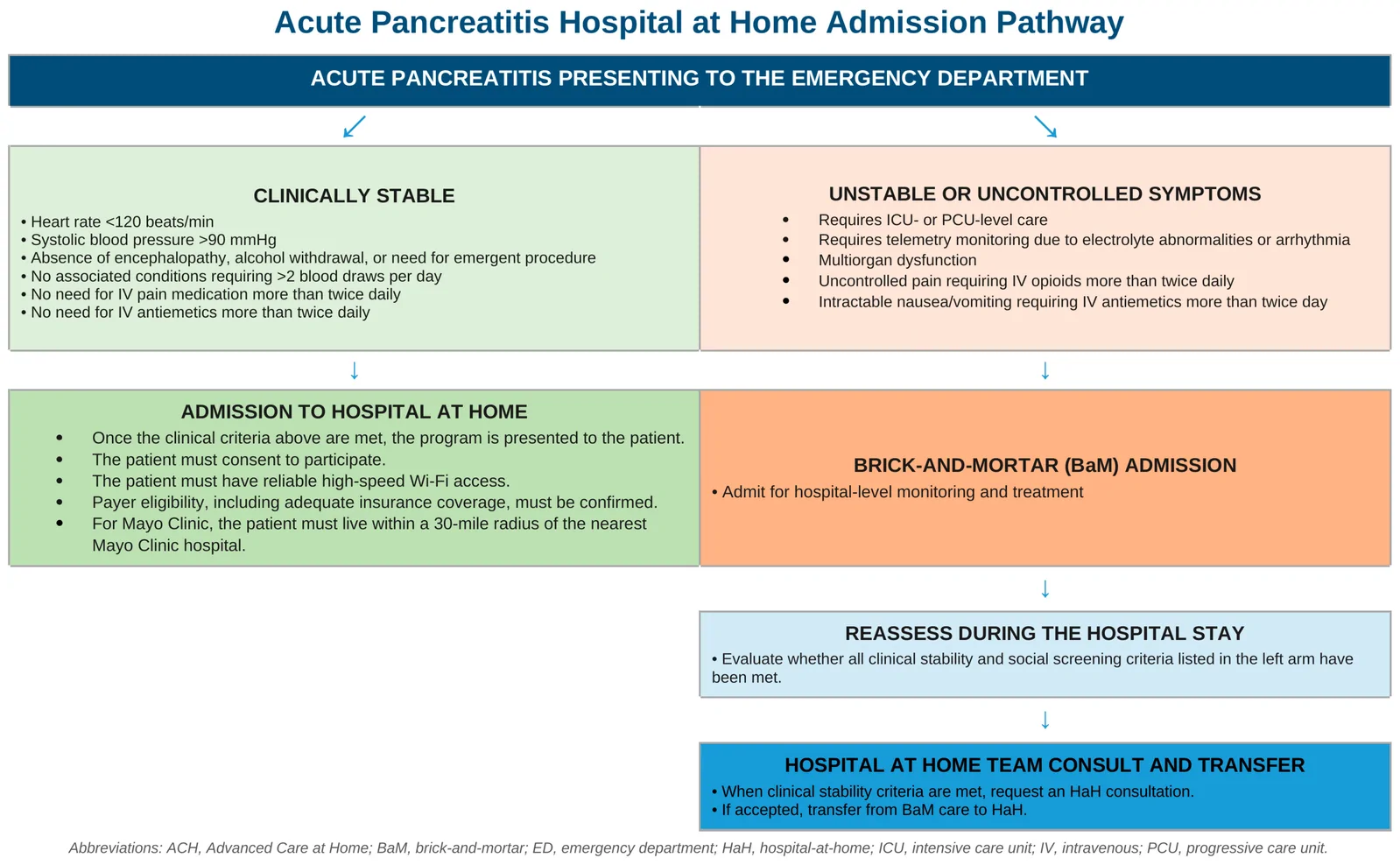
Acute pancreatitis Hospital-at-Home admission pathway and ACH eligibility screening. Color coding: Green boxes indicate the ACH pathway, orange boxes indicate brick-and-mortar hospital care or ACH exclusion criteria, and blue boxes indicate reassessment or transition decision points.

Patients may be admitted to ACH either directly from the Emergency Department, bypassing the traditional BaM hospital ward as hospital substitution model, or through transfer from a BaM hospital after initial stabilization. Patients meeting inpatient criteria for hospital admission are screened for ACH eligibility based on clinical stability, frequency of intravenous medications and laboratory testing, social factors, geographic location, insurance eligibility, and home safety (Figure 1).

For patients with pancreatitis receiving hospital-at-home care, any new or worsening symptoms may prompt immediate reassessment. Patients or their caregivers can initiate this process at any time by contacting the virtual nurse, who is available 24/7 through the iPad provided by the program. The virtual team evaluates new or worsening symptoms and coordinates in-person assessment when clinically indicated. Escalation of care was defined as any physical transfer from ACH to an ED or BaM hospital during the ACH episode, including both planned procedural transfers and unplanned transfers for clinical deterioration. Clinically stable patients requiring services beyond ACH capability were transferred directly to BaM care, whereas emergency medical services were activated for acute decompensation or instability. Patients who remained clinically stable and could be safely managed in the home setting continued ACH care with appropriate monitoring and treatment.

### 2.2. Study Design and Patient Population

This multicenter, multistate, retrospective descriptive cohort study included the index ACH admission of patients managed between 13 October 2020 and 1 June 2024, with a primary diagnosis of acute pancreatitis. The ACH program encompasses urban academic medical centers at the Mayo Clinic in Jacksonville, Florida (MCF) and the Mayo Clinic in Phoenix, Arizona (MCA), along with a program at rural northwest Wisconsin, affiliated with community teaching hospital at Mayo Clinic Health System (MCHS) in Eau Claire, Wisconsin (WI). Potential cases were identified using Diagnosis-Related Group codes 438, 439, and 440. Every identified chart underwent manual review. Acute pancreatitis was confirmed when at least two out of three criteria were present: characteristic abdominal pain, serum lipase or amylase levels at least three times upper limit of normal, or characteristic computed tomography (CT) findings on admission [1,2]. Chart review was performed by physician investigators (J.L. and B.J) and a research trainee (T.K.) under the supervision of the first author (T.G.). The initial dataset included 50 patient encounters. Repeat admissions for the same patient were excluded from the primary analysis, yielding a final cohort of 40 unique patients. Follow-up outcomes were assessed through the Mayo Clinic electronic health record for 30 days after discharge from the index admission. The retrospective chart review study was approved by the Mayo Clinic Institutional Review Board (protocol 25-004144).

### 2.3. Data Collection

Trained research interns and physicians extracted demographic and clinical data from the electronic health record. Variables collected included demographics (age, sex, race, and Mayo Clinic site), primary presenting symptoms, and pancreatitis etiology. Etiologies were categorized as gallstone-related, alcohol-related, idiopathic, post-procedure (including endoscopic retrograde cholangiopancreatography [ERCP]), medication-related, hypertriglyceridemia, anatomic (ampullary or biliary stenosis) or other. Medication-related etiology was classified according to the treating clinician’s attribution from treating provider or gastroenterology consultant’s notes, and a formal drug-causality assessment tool was not applied. The “other” category comprised cases of non-gallstone mechanical obstruction documented by the treating team.

Disease severity was assessed retrospectively, using the Ranson criteria on admission (initial presentation to ED) and at 48 h after admission irrespective of timing of ACH transfer [12], and the presence of systemic inflammatory response syndrome (SIRS).

Pancreatitis-related complications, present at any time during index admission, included: pulmonary edema, pulmonary infiltrates, necrotic or hemorrhagic pancreatitis, infected pancreatitis requiring intravenous antibiotic therapy, and peripancreatic fluid collections. Presence of SIRS and AKI was documented if either was identified at presentation to ED or within 48 h after admission.

Comorbidities included hypertriglyceridemia, alcohol use disorder, cholelithiasis, hypertension, and diabetes mellitus. Hydration, pain, and nausea management strategies were evaluated only during the active ACH stay following ED presentation or transfer from BaM stay to assess the feasibility of managing acute pancreatitis in the home setting. Treatment variables included non-opioid and opioid analgesics by route of administration, intravenous antiemetics, and intravenous fluid therapy categorized as bolus administration, continuous infusion, or combination of both. The type, volume and administration strategy of intravenous fluids were determined by the treating clinician according to individual patient needs.

Procedure-related escalations of care from ACH for acute pancreatitis management were documented and included ERCP, laparoscopic cholecystectomy, and chemical or endoscopic necrosectomy. These procedures were performed in BaM procedural settings. Safety outcomes included escalation of care to a BaM hospital, length of stay (LOS), 30-day emergency department visits, 7-day and 30-day readmissions, and 30-day mortality. Escalations were classified as either planned procedural transfers, transfer for care exceeding ACH capabilities or unplanned clinical deterioration. A 7-day readmission was defined as rehospitalization within 1 to 7 days of discharge, whereas the 30-day readmission outcome captured rehospitalizations occurring between days 8 and 30 after discharge.

### 2.4. Statistical Analysis

Descriptive statistics were calculated to summarize patient demographics, clinical characteristics, treatment variables, and outcomes for each ACH admission pathway. Continuous variables are presented as medians with interquartile ranges (IQR), as well as range. Categorical variables were reported as frequencies and percentages, and 95% confidence intervals for key clinical event rates were calculated using Clopper–Pearson exact methods. The analysis was carried out using R Statistical Software (version 4.4.1; R Foundation for Statistical Computing, Vienna, Austria).

## 3. Results

### 3.1. Clinical and Demographic Characteristics

The cohort comprised 40 unique patients with a median age of 67.5 years (Q1, Q3: 50.25, 75.25), of whom 23 (57.5%) were male and 35 (87.5%) were white (Table 1). Thirty-five patients (87.5%) entered ACH after a BaM stay, and five (12.5%) were admitted directly from the ED. Mayo Clinic Florida managed 17 patients (42.5%), Mayo Clinic Health System managed 12 (30.0%), and Mayo Clinic Arizona managed 11 (27.5%) (Table 1).

The primary presenting symptom was abdominal pain in 33 patients (82.5%), nausea or vomiting in 4 (10.0%), and another symptom in 3 (7.5%). The most common etiologies were idiopathic in 17 patients (42.5%), gallstone-related in 9 (22.5%), and alcohol-related in 5 (12.5%). Medication-induced pancreatitis included cases associated with pembrolizumab, pembrolizumab/axitinib, and semaglutide. SIRS was present at initial acute-care presentation in 3 patients (7.5%). Peripancreatic fluid collections occurred in 7 (17.5%), pleural effusions in 5 (12.5%), necrotizing pancreatitis in 4 (10.0%), and infected pancreatitis in 3 (7.5%). All patients with infected pancreatitis received IV antibiotics. Laboratory findings are summarized in Table 2.

At the initial acute-care presentation, all 40 patients had a Ranson score of 0–2. At 48 h, 38 patients (95.0%) had scores of 0–2 and 2 (5.0%) had scores of 3–4. CTSI categories were mild in 37 patients (92.5%), moderate in 1 (2.5%), and severe in 2 (5.0%) (Table 3). These scores preceded ACH entry for patients transferred after a BaM stay.

### 3.2. Healthcare Utilization and Clinical Outcomes

Of the 40 patients, 6 (15.0%) received only opioid analgesics, 11 (27.5%) received only non-opioid analgesics, 17 (42.5%) received both opioid and non-opioid analgesics, and 6 (15.0%) received neither. Intravenous antiemetics were administered to 9 patients (22.5%): ondansetron to 8 (20.0%), promethazine to 1 (2.5%), and prochlorperazine to 1 (2.5%); antiemetic categories were not mutually exclusive. Intravenous fluids were administered to 25 patients (62.5%): bolus only to 6 (15.0%), continuous infusion only to 8 (20.0%), and both to 11 (27.5%). Treatment details are shown in Table 4.

Median total LOS was 5.0 days (Q1, Q3: 3.0, 8.25; range, 2–24), including a median ACH LOS of 3.0 days (Q1, Q3: 2.0, 4.0; range, 1–12). Among the 35 patients transferred from BaM care, median pre-ACH BaM LOS was 2.0 days (Q1, Q3: 1.0, 3.5; range, 0–22). Escalation of care occurred in 4 patients: three planned pancreatitis-related procedural transfers, and one non-pancreatitis-related transfer for new diagnosis of advanced metastatic urothelial cancer and hospice care, accounting for the 2% 30-day mortality in this cohort, although death was unrelated to pancreatitis. No transfer was documented as resulting from unplanned clinical deterioration. There was no in-program mortality. No 30-day ED visits were observed. The 7-day and 30-day readmission rates were 10% and 5%, respectively. One patient was readmitted for bacteremia due to cholangitis in the setting of stable necrotizing pancreatitis with unchanged walled-off fluid collection. All other readmissions were due to acute on chronic pancreatitis without radiographic evidence of disease progression.

## 4. Discussion

To our knowledge, this is the first multicenter retrospective study that describes the characteristics, treatment requirements, and short-term outcomes of 40 unique patients with acute pancreatitis managed in a HaH setting from two admission pathways, either transfer from initial BaM care or directly from ED. This study describes the characteristics, treatment requirements and short-term outcomes of clinically stable patients with acute pancreatitis managed through the ACH program. Most patients in this cohort had mild pancreatitis. Notably, several patients with AKI, SIRS, infected pancreatitis or necrotizing pancreatitis were transferred to ACH after achieving clinical stability during initial BaM hospitalization, illustrating that selected patients with more complex initial disease presentation entered ACH after stabilization in BaM. Importantly, the entire cohort was closely monitored by 24/7 command center, with established processes in place to facilitate rapid escalation of care in the event of clinical deterioration or if the need for symptomatic management exceeded the capabilities of the ACH. Most patients required standard supportive therapies including pain management and IV hydration demonstrating feasibility of delivering treatment modalities per providers choice according to individual patient needs, including planned escalation back to the traditional BaM hospital for pancreatitis related procedures when indicated. Importantly, there were no escalations of care due to clinical instability, no in-program mortality, and cohort had low rates of emergency visits or readmissions. Accordingly, the findings describe feasibility within the ACH eligibility framework and do not establish comparative safety, effectiveness, superiority, or non-inferiority relative to conventional hospitalization.

The most common etiologies of pancreatitis in BaM include gallstones (40%), alcohol (30%), and hypertriglyceridemia (2–7%) [1,14]. The most common etiologies in this ACH cohort were idiopathic (42.5%), gallstone-related (22.5%) and alcohol-related (12.5%). Published acute pancreatitis studies provide clinical context but are not directly comparable because ACH eligibility excludes patients with instability or care needs that cannot be delivered at home [1,9,14]. Nevertheless, patients who achieved clinical stability during their initial inpatient hospitalization could subsequently be evaluated for transfer from BaM care to ACH for continuation of acute pancreatitis management. Although program-specific guidelines help identify appropriate candidates for ACH and promote patient and staff safety, each patient is assessed on an individual basis to determine ongoing suitability for home-based acute care. Significant alcohol use by patients may dissuade healthcare team members from enrolling patients to ACH, and BaM hospitalization might seem a safer model of care delivery. However, this process of patient identification is nuanced. Mitigating circumstances allow for the individualization of care and consideration of ACH for treatment of acute pancreatitis, even when alcohol use is the etiology of acute pancreatitis. To ensure patient and staff safety, ACH does not enroll patients when there is concern for alcohol withdrawal, electrolyte disturbances and/or need for telemetry, which may explain why there are fewer patients with alcohol-induced pancreatitis in HaH compared to BaM.

Risk factors for the development of severe pancreatitis are elevated blood urea nitrogen, hematocrit, any comorbid medical condition, and the presence of SIRS [3]. Despite having multiple different scoring models for severity, the presence of SIRS, Revised Atlanta Classification systems, and Determinant-Based Classification systems are recommended for predicting severity of pancreatitis; however, scoring systems have limited clinical value and should not be guidance for management [14,15,16]. Ranson and CTSI values in this study refer to the initial acute-care presentation, which preceded ACH transfer for 35 patients. Two patients had a severe CTSI category based on initial imaging, but entry into ACH occurred only after the program’s clinical stability criteria were met. CTSI reflects morphologic findings and does not by itself establish persistent organ failure at ACH entry.

The outcome estimates are descriptive. Escalation of care from ACH to BaM for acute pancreatitis of 10% was for pancreatitis-related procedures (necrosectomy, cholecystectomy and ERCP), but also for treatment of other medical problems. For context, previously reported escalation of care in HaH for acute decompensated heart failure ranged from 10.3% to 15.98% [17,18]. Median total ACH LOS was 5 days, comprising a median of 2.0 days in BaM care before ACH transfer and 3.0 days in ACH. Reportedly, LOS for acute pancreatitis in a traditional hospital setting was 6.99 days in 2002 and decreased to 5.74 days in 2013 [19]. The length of stay for other conditions treated in HaH range from 3.6 to 5.7 days depending on diagnosis [20,21]. Among patients with acute pancreatitis managed in the Mayo Clinic ACH program, no 30-day ED visits were observed and the 30-day readmission rate was 5%. Previously published Mayo Clinic ACH studies across all diagnoses reported a 15% 30-day ED visit rate and a 14% 30-day readmission rate [6]. For context only, reported readmission rates after traditional hospital admission for acute pancreatitis can range from 7 to 34%, varying by etiology and severity and presence of necrosis [22]. In our program, 30-day all-cause mortality following discharge from ACH for acute pancreatitis admission was 2.5%, while in-program mortality was 0%. National mortality rates for acute pancreatitis were reported at 2.85% in 2008 and 2.01% in 2015 [23]. Prior HaH studies have reported favorable outcomes with HaH care, including reduced mortality, readmissions, and costs, as well as improved patient satisfaction in selected populations. Mayo Clinic’s randomized trial also demonstrated noninferior 30-day readmission and mortality outcomes for ACH compared with traditional hospitalization. These findings provide clinical context only and are not directly comparable to the present cohort because of differences in patient populations and study design [6,24,25].

### Strengths and Limitations

Our study has several strengths. To our knowledge, it is the first multi-site descriptive retrospective study of acute pancreatitis managed in a HaH program spanning both academic and community settings, and it provides patient-level descriptions of admission pathway, comorbidities, laboratory findings, treatment patterns and outcomes.

This study has several limitations. This study was limited by the inclusion of only clinically stable patients, which may restrict the generalizability of the findings to individuals with critical illness or multi-organ failure. A key limitation of this study is the inability to quantify the full denominator of patients with acute pancreatitis evaluated for potential ACH admission during the study period, the total number of acute pancreatitis hospitalizations, patients screened for HaH eligibility, those deemed ineligible, and those who declined participation were not systematically available. Consequently, the degree of selection and potential selection bias cannot be fully assessed, which may limit the generalizability of the findings to the broader population of patients hospitalized with acute pancreatitis. Furthermore, because follow-up data were derived from the electronic health record, emergency department visits or hospitalizations occurring outside the Mayo Clinic system may not have been captured, which could have led to under-ascertainment of 30-day healthcare utilization outcomes. Revised Atlanta and determinant-based categories were not reconstructed because organ failure duration and timing relative to ACH entry were not consistently captured. Patient-level physiologic and treatment data at the exact time of ACH entry were not uniformly available, limiting characterization of clinical status at transfer. Next, the relatively small cohort size and retrospective design limit statistical power. Additionally, all patient encounters were classified as inpatient status, which may limit comparison to observation models of pancreatitis care. Costs and comparative resource use were not evaluated. Future studies incorporating contemporaneous, eligibility-matched comparison groups are needed to evaluate comparative clinical outcomes and healthcare utilization.

## 5. Conclusions

Among carefully selected patients meeting the program’s eligibility criteria, management of acute pancreatitis in HaH was feasible and was associated with low observed rates of unplanned escalation and short-term adverse outcomes.

## Figures and Tables

**Table 1:** Demographic and clinical characteristics by ACH admission pathway in the 40-patient cohort.

Variable	BaM Transfer (N = 35)	Direct ED (N = 5)	Total (N = 40)
Demographic			
Age, years, median (Q1, Q3); range	70.00 (51.50, 76.00); 36–91	51.00 (43.00, 51.00); 36–68	67.50 (50.25, 75.25); 36–91
Male sex	19 (54.3%)	4 (80.0%)	23 (57.5%)
ACH site			
Arizona	11 (31.4%)	0 (0.0%)	11 (27.5%)
Florida	14 (40.0%)	3 (60.0%)	17 (42.5%)
Mayo Clinic Health System, Wisconsin	10 (28.6%)	2 (40.0%)	12 (30.0%)
Race			
African American	1 (2.9%)	1 (20.0%)	2 (5.0%)
Native Hawaiian/Pacific Islander	0 (0.0%)	1 (20.0%)	1 (2.5%)
Other/unknown	2 (5.7%)	0 (0.0%)	2 (5.0%)
White	32 (91.4%)	3 (60.0%)	35 (87.5%)
Etiology			
Gallstone-related	9 (25.7%)	0 (0.0%)	9 (22.5%)
Alcohol-related	5 (14.3%)	0 (0.0%)	5 (12.5%)
Other/mechanical obstruction	1 (2.9%)	0 (0.0%)	1 (2.5%)
Idiopathic	13 (37.1%)	4 (80.0%)	17 (42.5%)
Medication induced	3 (8.6%)	0 (0.0%)	3 (7.5%)
Hypertriglyceridemia	2 (5.7%)	1 (20.0%)	3 (7.5%)
Post-ERCP procedure	2 (5.7%)	0 (0.0%)	2 (5.0%)
Primary presenting symptom			
Abdominal pain	28 (80.0%)	5 (100.0%)	33 (82.5%)
Nausea/vomiting	4 (11.4%)	0 (0.0%)	4 (10.0%)
Other	3 (8.6%)	0 (0.0%)	3 (7.5%)
Associated findings			
SIRS at initial presentation	3 (8.6%)	0 (0.0%)	3 (7.5%)
Acute kidney injury	8 (22.9%)	0 (0.0%)	8 (20.0%)
Peripancreatic fluid collection	7 (20.0%)	0 (0.0%)	7 (17.5%)
Pleural effusion	5 (14.3%)	0 (0.0%)	5 (12.5%)
Necrotizing pancreatitis	4 (11.4%)	0 (0.0%)	4 (10.0%)
Infected pancreatitis	3 (8.6%)	0 (0.0%)	3 (7.5%)

Continuous variables are reported as median (Q1, Q3) and range; categorical variables are reported as n (%). BaM transfer denotes ACH admission after initial brick-and-mortar hospital care; direct ED denotes direct ACH admission from the emergency department. ACH, Advanced Care at Home; BaM, brick-and-mortar; ED, emergency department; ERCP, endoscopic retrograde cholangiopancreatography; SIRS, systemic inflammatory response syndrome. Background colors are used solely to visually distinguish workflow components and do not indicate differences in patient status, eligibility, or outcomes. “N” represents the sample size.

**Table 2:** Comorbidities and laboratory findings in the pancreatitis cohort managed in the Mayo Clinic ACH program.

Variable	ACH (N = 40)
Comorbidities	
Hyperlipidemia/hypercholesterolemia	25 (62.5%)
Diabetes mellitus	9 (22.5%)
Hypertriglyceridemia	7 (17.5%)
Alcohol use disorder	4 (10.0%)
Cirrhosis	2 (5.0%)
Cholelithiasis	3 (7.5%)
Hypertension	1 (2.5%)
Laboratory findings at admission	
WBC, ×10^9^/L (3.4–9.6)	7.75 (5.20, 11.13); 3.20–26.00
Hemoglobin, g/dL (13.2–16.0)	11.75 (10.38, 13.33); 7.80–15.80
Platelets, ×10^9^/L (135–317)	210.00 (173.00, 279.00); 130.00–672.00
Creatinine, mg/dL (0.74–1.35)	0.86 (0.73, 1.03); 0.48–2.59
Sodium, mmol/L (135–145)	137.00 (136.00, 139.25); 120.00–146.00
AST, U/L (8–48)	25.50 (21.00, 42.00); 12.00–1442.00
ALT, U/L (7–55)	23.00 (15.00, 37.25); 8.00–1211.00
Albumin, g/dL (3.5–5.0)	3.45 (3.15, 4.03); 1.90–4.60
Triglycerides, mg/dL (<150)	131.00 (94.00, 175.00); 56.00–427.00
CRP, mg/L (<5.0)	52.25 (32.68, 71.05); 6.20–190.90
Lactate, mmol/L (0.5–2.0)	1.40 (1.00, 1.73); 0.70–8.60
Lipase, U/L (13–60)	203.00 (91.25, 656.75); 13.00–18,080.00
Glucose, mg/dL (70–140)	112.50 (93.00, 127.25); 74.00–150.00

Continuous laboratory variables are reported as median (Q1, Q3); range. Categorical variables are reported as n (%). Adult reference intervals in the variable labels are representative Mayo Clinic Laboratories intervals and may vary by assay, testing site, and sex [13]. WBC, white blood cell count; AST, aspartate aminotransferase; ALT, alanine aminotransferase; CRP, C-reactive protein. “N” represents the sample size. Background colors are used solely to visually distinguish workflow components and do not indicate differences in patient status, eligibility, or outcomes.

**Table 3:** Ranson and computed tomography severity scores by ACH admission pathway.

Variable	BaM Transfer (N = 35)	Direct ED (N = 5)	Total (N = 40)
Ranson score at initial presentation			
Median (Q1, Q3)	1.00 (0.50, 1.00)	0.00 (0.00, 1.00)	1.00 (0.00, 1.00)
Range	0–2	0–1	0–2
Mild (0–2)	35 (100.0%)	5 (100.0%)	40 (100.0%)
Moderate (3–4)	0 (0.0%)	0 (0.0%)	0 (0.0%)
Severe (≥5)	0 (0.0%)	0 (0.0%)	0 (0.0%)
Ranson score at 48 h			
Median (Q1, Q3)	2.00 (1.00, 2.00)	1.00 (0.00, 1.00)	2.00 (1.00, 2.00)
Range	0–3	0–2	0–3
Mild (0–2)	33 (94.3%)	5 (100.0%)	38 (95.0%)
Moderate (3–4)	2 (5.7%)	0 (0.0%)	2 (5.0%)
Severe (≥5)	0 (0.0%)	0 (0.0%)	0 (0.0%)
CTSI at initial presentation			
Median (Q1, Q3)	2.00 (1.50, 3.00)	2.00 (2.00, 2.00)	2.00 (1.75, 3.00)
Range	0–10	0–3	0–10
Mild (0–3)	32 (91.4%)	5 (100.0%)	37 (92.5%)
Moderate (4–6)	1 (2.9%)	0 (0.0%)	1 (2.5%)
Severe (≥7)	2 (5.7%)	0 (0.0%)	2 (5.0%)

Scores refer to the initial presentation to ED and after 48 h of admission. Ranson and CTSI data were available for 40/40 patients. The Ranson criteria is a scoring system that uses eleven prognostic indicators suggestive of the development of severe acute pancreatitis where each indicator is assigned a point value, and ranges of values have associated different mortality rate (0–2 points, mortality is 0% to 3%; 3–4 points, mortality is 15%; 5–6 points, mortality is 40%; and for 7–11 points, mortality approaches 100%) (8). CTSI, computed tomography severity index. “N” represents the sample size. Background colors are used solely to visually distinguish workflow components and do not indicate differences in patient status, eligibility, or outcomes.

**Table 4:** Management strategies, utilization and outcomes by ACH admission pathway.

Variable	BaM Transfer (N = 35)	Direct ED (N = 5)	Total (N = 40)
Analgesic exposure, mutually exclusive			
Only opioid analgesic	6 (17.1%)	0 (0.0%)	6 (15.0%)
Only non-opioid analgesic	8 (22.9%)	3 (60.0%)	11 (27.5%)
Both opioid and non-opioid analgesics	15 (42.9%)	2 (40.0%)	17 (42.5%)
Neither opioid nor non-opioid analgesic	6 (17.1%)	0 (0.0%)	6 (15.0%)
Non-opioid analgesics			
Any non-opioid analgesic	23 (65.7%)	5 (100.0%)	28 (70.0%)
Oral administration	23 (65.7%)	2 (40.0%)	25 (62.5%)
Acetaminophen	23 (65.7%)	2 (40.0%)	25 (62.5%)
Gabapentin	2 (5.7%)	0 (0.0%)	2 (5.0%)
Ibuprofen	2 (5.7%)	1 (20.0%)	3 (7.5%)
Methocarbamol	1 (2.9%)	0 (0.0%)	1 (2.5%)
Intravenous administration	4 (11.4%)	4 (80.0%)	8 (20.0%)
Ketorolac	4 (11.4%)	4 (80.0%)	8 (20.0%)
Opioid analgesics			
Any opioid analgesic	21 (60.0%)	2 (40.0%)	23 (57.5%)
Oral administration	21 (60.0%)	1 (20.0%)	22 (55.0%)
Oxycodone	15 (42.9%)	1 (20.0%)	16 (40.0%)
Hydromorphone	4 (11.4%)	0 (0.0%)	4 (10.0%)
Hydrocodone/acetaminophen	2 (5.7%)	0 (0.0%)	2 (5.0%)
Intravenous administration	2 (5.7%)	1 (20.0%)	3 (7.5%)
Fentanyl	1 (2.9%)	1 (20.0%)	2 (5.0%)
Hydromorphone	1 (2.9%)	0 (0.0%)	1 (2.5%)
Intravenous antiemetics			
Any intravenous antiemetic	8 (22.9%)	1 (20.0%)	9 (22.5%)
Ondansetron	7 (20.0%)	1 (20.0%)	8 (20.0%)
Promethazine	1 (2.9%)	0 (0.0%)	1 (2.5%)
Prochlorperazine	1 (2.9%)	0 (0.0%)	1 (2.5%)
Intravenous fluid administration			
Any intravenous fluid	20 (57.1%)	5 (100.0%)	25 (62.5%)
Bolus and continuous infusion	8 (22.9%)	3 (60.0%)	11 (27.5%)
Bolus only	4 (11.4%)	2 (40.0%)	6 (15.0%)
Continuous infusion only	8 (22.9%)	0 (0.0%)	8 (20.0%)
No intravenous fluid	15 (42.9%)	0 (0.0%)	15 (37.5%)
Length of stay			
BaM LOS before ACH transfer, days, median (Q1, Q3); range	2.00 (1.00, 3.50); 0–22	NA	2.00 (1.00, 3.50); 0–22
ACH LOS, days, median (Q1, Q3); range	2.00 (2.00, 4.00); 1–12	3.00 (3.00, 4.00); 2–4	3.00 (2.00, 4.00); 1–12
Total ACH LOS, days, median (Q1, Q3); range	5.00 (4.00, 9.00); 2–24	3.00 (3.00, 4.00); 3–4	5.00 (3.00, 8.25); 2–24
Short-term outcomes			
Escalation of care to BaM	4 (11.4%; 95% CI, 3.2–26.7%)	0 (0.0%; 95% CI, 0.0–52.2%)	4 (10.0%; 95% CI, 2.8–23.7%)
7-day readmission	3 (8.6%; 95% CI, 1.8–23.1%)	1 (20.0%; 95% CI, 0.5–71.6%)	4 (10.0%; 95% CI, 2.8–23.7%)
30-day readmission	2 (5.7%; 95% CI, 0.6–19.2%)	0 (0.0%; 95% CI, 0.0–52.2%)	2 (5.0%; 95% CI, 0.6–16.9%)
30-day ED visit	0 (0.0%; 95% CI, 0.0–10.0%)	0 (0.0%; 95% CI, 0.0–52.2%)	0 (0.0%; 95% CI, 0.0–8.8%)
30-day mortality	1 (2.9%; 95% CI, 0.0–14.9%)	0 (0.0%; 95% CI, 0.0–52.2%)	1 (2.5%; 95% CI, 0.0–13.2%)
Pancreatitis-related procedures			
Any pancreatitis-related procedure	3 (7.5%)	0 (0.0%)	3 (7.5%)
ERCP	1 (2.9%)	0 (0.0%)	1 (2.5%)
Endoscopic necrosectomy	1 (2.9%)	0 (0.0%)	1 (2.5%)
Laparoscopic cholecystectomy	1 (2.9%)	0 (0.0%)	1 (2.5%)

Continuous variables are reported as median (Q1, Q3) and range; categorical variables are reported as n (%). Medication-specific and antiemetic categories may overlap; the four analgesic-exposure categories and four intravenous-fluid categories are mutually exclusive. Confidence intervals are exact Clopper–Pearson 95% CIs. ACH, Advanced Care at Home; BaM, brick-and-mortar; CI, confidence interval; ED, emergency department; ERCP, endoscopic retrograde cholangiopancreatography; LOS, length of stay; NA, not applicable. “N” represents the sample size. Background colors are used solely to visually distinguish workflow components and do not indicate differences in patient status, eligibility, or outcomes.

## Data Availability

The data presented in this study are available from the corresponding author upon reasonable request. The data are not publicly available due to patient privacy considerations and institutional restrictions.

## Abbreviations

ACH—Advanced Care at Home, HaH—Hospital at Home, BaM—Brick-and-Mortar, MCF—Mayo Clinic Florida, MCA—Mayo Clinic Arizona, MCHS/NWWI—Mayo Clinic Health System—Northwest Wisconsin, CMS—Centers for Medicare & Medicaid Services, DRG—Diagnosis-Related Group, ERCP—Endoscopic Retrograde Cholangiopancreatography, SIRS—Systemic Inflammatory Response Syndrome, CTSI—Computed Tomography Severity Index, LOS—Length of Stay, ED—Emergency Department, PO—Per Oral, IV—Intravenous.
